# Talking the Talk, or Walking the Walk? How Managerial Practices Relate to Nonprofit Organizations’ Role as Schools of Democracy

**DOI:** 10.1177/08997640241278634

**Published:** 2024-09-20

**Authors:** Lisa Hohensinn, Julia Litofcenko, Florentine Maier, Leila Cornips

**Affiliations:** 1 WU Vienna University of Economics and Business, Austria

**Keywords:** nonprofit organizations, becoming business-like, managerialization, organizational democracy, participation, political engagement, civic engagement

## Abstract

Nonprofit organizations are touted as “schools of democracy” that teach civic skills and values, but their increasing use of managerial practices from the business world may endanger this role. We examine the relationships between nonprofits’ managerial practices, practices of organizational democracy, and endorsement of public participation. Using organizational-level survey data from the Viennese metropolitan area, we find that the extent to which nonprofits use managerial practices negatively relates to their degree of organizational democracy. However, greater use of democratic and managerial practices positively relates to endorsing public participation. We conclude that managerialization often turns nonprofits from practitioners into mere preachers of democracy.

## Introduction

As political-administrative systems in democratic countries have been struggling with legitimacy problems for quite some time (Van Beek, 2018), nonprofit organizations are often looked to as beacons of hope. They are often understood as mediating institutions between citizens and government (LeRoux, 2007) that fulfill an important societal role by promoting civic engagement (Moulton & Eckerd, 2012). In the Tocquevillian tradition, this role has been called “schools of democracy” or schools of citizenship (de Tocqueville, 2010; Putnam, 1995). Nonprofit organizations that serve as schools of democracy produce citizens who are willing and able to participate fully in society through nonviolent means (Dodge & Ospina, 2016; Fung, 2003; Jo, 2020).

Yet, uncertainties persist about the role of nonprofit organizations as schools of democracy (Dekker, 2014; Van Ingen & Van der Meer, 2016). While we know that members’ or volunteers’ engagement in nonprofit organizations is positively correlated with their public participation (Howard & Gilbert, 2008; Lee, 2020; Van der Meer & Van Ingen, 2009; Van Ingen & Van der Meer, 2016), there is still a dearth of evidence, especially in quantitative terms, on what kinds of organizational practices facilitate or hinder nonprofit organizations’ contributions as schools of democracy (Dodge & Ospina, 2016; Van der Meer & Van Ingen, 2009). More research is hence needed to unpack the conditions of nonprofits’ functioning as schools of democracy (Dekker, 2014, p. 56; Eikenberry, 2022, p. 113).

Scholars have expressed concern that the increasing managerialization (Hvenmark, 2016) and becoming business-like of nonprofit organizations is jeopardizing their role in promoting democracy (see, for example, Eikenberry & Kluver, 2004; Frumkin, 2002; King & Griffin, 2019). These phenomena can be analyzed from a variety of perspectives (Hvenmark, 2016), focusing on processes, ideologies, or practices.

We adopt a practice perspective and hence use the concept of managerial practices (Brandtner, 2021) to take specific techniques and routinized actions into focus. “[. . .P]ractices have a virtual existence as largely unconscious yet shared and recognizable ways of doing things.” (Jarzabkowski et al., 2016, p. 271). Managerial practices are the manifestation of a managerial institutional logic or market logic (Skelcher & Smith, 2015). As practices, they complement the latent dimension of managerialism as an ideology (Hvenmark, 2016). Which practices reflect managerialism necessarily varies over time and somewhat from context to context (e.g., having a “mission statement” is a common practice for nonprofits in the United States, but for reasons of signaling language a distinctly managerial practice in Austria). Managerialism is a dynamic ideology that has evolved from idealizing steep hierarchies and direct control to more sophisticated notions that include promises of participation, self-actualization, and egalitarianism (Costea et al., 2008). Despite these variations and complexities, there is some basic agreement in research on what types of practices might currently be considered typical (e.g., Brandtner, 2021; Hersberger-Langloh et al., 2021; Suykens et al., 2023; Xu, 2024): strategic planning, working with management consultants, hiring professional managers, performance-based pay, and the like. Managerial practices also include governance practices such as having external audits (e.g., Brandtner, 2021) and policy-making boards (Maier & Meyer, 2011), which are supposed to foster transparency and accountability.

The adoption of managerial practices has been shown in several qualitative studies to weaken the pro-democratic role of nonprofits by promoting passive subjectivities as consumers and clients (Baines et al., 2011; Hvenmark, 2013; Skocpol, 2003; Tatarchevskiy, 2011; Wirgau et al., 2010). In contrast, participatory and empowering organizational practices that position individuals who engage in nonprofits as critical and collectively engaged citizens have been shown to promote democratic skills and attitudes (Dodge & Ospina, 2016). This is in line with the spillover effect from organizational democracy to pro-democratic values that have been found in for-profit businesses (Weber et al., 2020).

We delve deeper into the examination of organizational practices and nonprofits’ role as schools of democracy by dividing this role into two parts: practicing organizational democracy and endorsing public participation.

Practicing organizational democracy means that nonprofits serve as schools of democracy by internally organizing democratically (King & Griffin, 2019). Specifically, it means that members participate in decision-making and governance, thereby shaping the direction of the organization (Harrison & Freeman, 2004). We understand organizational democracy as a particular form of participatory governance (Wichowsky et al., 2023), in which organizational members not only have the opportunity to express their views but also have substantial power to influence tactical and strategic organizational decisions (Unterrainer et al., 2011). As with managerialism and management practices, ideals of organizational democracy and the corresponding practices vary somewhat over time and across contexts. For example, democratic organizations can be more or less hierarchical (Battilana et al., 2022) and can make their collective decisions using a variety of procedures (for instance, majority voting or consensus, Maier & Meyer, 2011).

Endorsement of public participation means that nonprofits encourage individuals to participate in politics, for example, by asking them to sign petitions, vote in elections, boycott products, or contact politicians. It refers to “the external role nonprofits play in producing democracy, particularly by engendering civic action” (King & Griffin, 2019, p. 910).

As we aim for a more nuanced understanding of the relationship between managerial practices and these two ways of being a school of democracy, we ask the following research question: How are nonprofit organizations’ use of managerial practices, their use of democratic organizing practices, and their endorsement of public participation related to each other? To answer this question, we use survey data from a random sample of nonprofit organizations in the Viennese metropolitan area and implement a path model with maximum likelihood estimation.

This article makes two contributions. First, we contribute empirical evidence to the long-running debate on the potential negative societal consequences of nonprofit organizations adopting business-like management methods (Maier et al., 2016; Suykens et al., 2019). While in the 1990s and early 2000s this debate was mainly based on conceptual arguments (e.g., Bush, 1992; Eikenberry & Kluver, 2004), a sustained stream of qualitative studies has since shed more light on the negative consequences (such as Baines et al., 2011; Hustinx & De Waele, 2015; Keevers et al., 2012; Kreutzer & Jager, 2011; Skocpol, 2003). They have been complemented by a growing number of well-designed quantitative studies, which have, however, refuted many critical arguments about negative consequences, and even provided contrary evidence (e.g., Hersberger-Langloh et al., 2021; Shirinashihama, 2019; Suykens et al., 2023). However, these quantitative studies have not looked at the impact of management practices on the transmission of pro-democratic values and skills. Our study provides quantitative data on this important issue, enabling a more factual debate on the weakening of organizational democracy, the diminished transmission of civic skills and values, and ultimately the erosion of democracy at the state level, which may be some of the more worrying concomitants of managerialization.

The second contribution is theoretical. By distinguishing between two different ways in which nonprofits can serve as schools of democracy—by organizing internally in democratic ways and by encouraging their members to engage in political action outside the organization—we arrive at the nuanced empirical result that managerialized organizations increase their endorsement of public participation while at the same time decreasing the practice of democracy in their own domain. This seemingly contradictory result prompts us to search for an explanation, and tease out a key difference between organizational democracy and dominant understandings of democracy at the state level: democracy as a means of overcoming individualism or as a means of enabling individualism. This difference in the meaning of democracy may explain why, when it comes to internal affairs of the organization, managerialism tends to deal with differences through transactional exchanges rather than through discursive deliberations that embrace diverse viewpoints and value them as contributions to new collective visions.

We develop our argument by first elaborating on the notion of nonprofit organizations as schools of democracy, summarizing the state of research on the effects of managerial practices on democracy, and formulating our hypotheses. We then describe our research context, methods of data collection and analysis. After presenting our findings, we close with a discussion of scholarly and practical implications, especially about the need to reconcile management tool use and organizational democracy to safeguard nonprofit organizations’ role as hands-on schools of democracy while still reaping the benefits of professional management.

## Theoretical Background and Hypotheses

According to the Neo-Tocquevillian approach, nonprofit organizations can be schools of democracy: places for citizens to learn civic skills and attitudes (Fung, 2003), express personal values and beliefs (Jo, 2020), generate trust and social bonds through face-to-face contact, and increase tolerance for diverse perspectives (de Tocqueville, 2010; Dodge & Ospina, 2016; Fung, 2003; Putnam, 1995, 2000). Instilling these civic virtues is known to be a prerequisite for a political culture that sustains democratic systems (Dodge & Ospina, 2016, p. 479).

Schools of democracy are assumed to promote political engagement because they “produce citizens able and ready to participate in society” (Dodge & Ospina, 2016, p. 479). While there is a self-selection effect of people with pro-democratic attitudes being more likely to engage in nonprofit organizations (Van Ingen & Van der Meer, 2016), the organizations can create “participatory spillover effects” (Karreth, 2018, p. 159) that mobilize members to participate in political life.

A similar argument has been made for for-profit businesses, which by adopting participatory modes of governance may create a spillover effect at the societal level (Pateman, 1970), as employees develop civic skills and attitudes that eventually play out in the public sphere at large (see Weber et al., 2020 for a meta-analysis; Timming & Summers, 2020 for further recent evidence). The thesis about nonprofit organizations as schools of democracy does not denigrate the possibility of a democratic spillover effect in for-profit businesses. It originates from de Tocqueville’s thought that while individuals need capital to participate in decision-making in a business context, all individuals would have equal access to participate in nonprofit organizations. Having in mind the experiences of business entrepreneurs and investors, he argued: “You can in general become familiar with the theory of association only by risking your money. Associations are the free schools of association” (de Tocqueville, 2010, p. 911, originally published in 1835), or in other words: In a business context, only wealthy people can participate in ways that allow them to develop political skills, while in nonprofit organizations, everybody can participate and learn such skills.

In the meantime, much has happened to put the perpetual wisdom of this thought about early 19th-century U.S. society into question, such as workplaces becoming more participatory (Boltanski & Chiapello, 2018), nonprofit organizations becoming less participatory (Skocpol, 2003), and empirical research showing that nonprofit organizations are not equally open to people from all walks of life (M. Meyer & Rameder, 2022). In the context of this article, we neither can nor want to examine whether nonprofit organizations or for-profit businesses (or other social systems such as families or schools) are the most important schools of democracy. Rather, we are interested in the causal mechanism that both streams of literature—on nonprofit organizations and businesses—understand in the same way: By being exposed to and applying democratic skills and virtues in an organizational context, individuals become more skillful and convinced democratic citizens at the state level as well.

While in research on businesses, scholars have been clear about the fact that businesses vary in their degree of organizational democracy, and spillover effects can only be expected in proportion to the degree of organizational democracy (Weber et al., 2020), research on nonprofit organizations has often assumed that nonprofits generally have pro-democratic effects. However, the scarce empirical research has not shown an across-the-board effect but rather that the extent to which organizations fulfill this educational role can vary (Dekker, 2014; Eikenberry, 2022; Kamstra & Knippenberg, 2014).

To enable a more nuanced analysis of the nonprofit’s role as schools of democracy, we divide this role into two aspects (King & Griffin, 2019): organizational democracy, and endorsement of public participation. On the one hand, nonprofits may serve as schools of democracy by internally organizing in democratic ways. Members, volunteers, employees, beneficiaries, or other stakeholders participate in organizational decision-making and thereby learn democratic values and skills through practice and direct experience (Dodge & Ospina, 2016; Foley & Polanyi, 2006). For example, by collectively deciding on important issues facing their organization and reconciling the interests and capabilities of different stakeholders, members can gain deep insight into how society as a whole might meet its collective challenges. Staying with the school metaphor, this mechanism is the hands-on learning method for democracy, or in other words, walking the walk of democracy. On the other hand, endorsement of public participation means that nonprofit organizations encourage stakeholders to participate in politics. For many nonprofits engaged in advocacy, this is a major part of their advocacy techniques. For example, nonprofits may exhort members to participate in national elections, and these mobilization efforts have been shown to significantly increase voter turnout (LeRoux et al., 2023). In the school of democracy metaphor, this would be the teaching method of direct instruction, or in other words, talking the talk of democracy. Thereby, we draw a line between nonprofits’ preaching of public participation, and their practice of democracy within their own domain.

Many scholars have expressed concern that as nonprofit organizations adopt more managerial practices from the business world, this jeopardizes their role as schools of democracy (see., e.g., Frumkin, 2002; Maier et al., 2016). To better understand the lines of reasoning behind these concerns and develop hypotheses for empirical analysis, we break the problem down into three parts: (a) the relationship between managerial practices and practices of organizational democracy, (b) the relationship between managerial practices and the endorsement of public participation, and (c) the relationship between practices of organizational democracy and the endorsement of public participation.

### Managerial Practices and Organizational Democracy

Managerial practices are widely understood to conflict with practices of organizational democracy. From the perspective of managerialist logic, having various stakeholder groups participate in democratic decision-making is of no intrinsic value, but is valuable only insofar as it helps the organization achieve its goals more efficiently and effectively (Kerr, 2004; Maier & Meyer, 2011). However, many benefits of organizational democracy occur not at the organizational, but rather at the individual level (e.g., higher employee satisfaction, for details see Weber et al., 2020) or societal level (e.g., the spillover or schools of democracy effect). The narrow business case for organizational democracy is not overwhelming (Harrison & Freeman, 2004); a direct, positive link between democratic forms of organizing and organizational performance is hard to establish. Moreover, as external stakeholders such as government funders, donors, or social investors increasingly expect nonprofits to be professional in a way similar to businesses, nonprofit organizations often cannot escape the pressure to adopt more managerial practices.

When attempting to combine managerial with democratic ways of organizing, however, nonprofits tend to face internal tensions (Baines et al., 2011; Hvenmark, 2013; Kreutzer & Jager, 2011). Although a combination of managerial and democratic practices is in principle possible (Maier & Meyer, 2011) and may even be productive (Skelcher & Smith, 2015), those different sets of practices are understood to belong to different institutional logics (Knutsen, 2012). Actors hence lack readily available templates for how to combine them.

In the wake of New Public Management reforms, nonprofit organizations have been driven toward more standardization, management control, and quantification of performance measurement, at the cost of unquantifiable practices in the relational, equity- and equality-focused, and participatory dimensions (Baines & Cunningham, 2020). Accordingly, several qualitative studies have found that these reforms have pushed nonprofit organizations to become ever more efficient and that, as a result, nonprofits have reduced or even eliminated practices of organizational democracy (Baines & Cunningham, 2020; Baines et al., 2011; Cunningham & James, 2014; Frumkin & Galaskiewicz, 2004). But even without such funding-related pressures, nonprofits have traded member-centered democratic forms of organizing for managerial ones in search of efficiency and effectiveness (Skocpol, 2003). We therefore hypothesize:

**Hypothesis 1 (H1):** The use of managerial practices is negatively related to organizational democracy.

### Managerial Practices and the Endorsement of Public Participation

Regarding the relationship between nonprofit organizations’ use of managerial practices and their endorsement of public participation, there has been less scholarly debate. Endorsement of public participation is widely agreed to be a part of nonprofit organizations fulfilling their societal roles (Fung, 2003; Moulton & Eckerd, 2012) and especially a part of fulfilling their advocacy role to “influence the decisions of any institutional elite on behalf of a collective interest” (Jenkins, 1987, p. 297).

Critical management scholars have argued that as nonprofit organizations become more business-like, this jeopardizes democracy at the state level. The mechanism they have pointed to as a reason for this negative effect is marketization (Eikenberry & Kluver, 2004; Sandberg et al., 2020): the adoption of the values, logic, and practices of a marketplace, where all things are commensurate in monetary terms, and actors relate to each other in competitive transactional ways while pursuing their individual interests. The prevalence of this worldview in mainstream management knowledge, they argue, erodes the foundations of state-level democracy in the long run (Eikenberry & Kluver, 2004).

Research from the perspectives of institutional theory and world polity sheds light on why, despite this ultimate contradiction with potentially fatal consequences, managerialist NPOs cannot be assumed to engage less in democratic discourse at the state level. On the contrary, they can be expected to engage vigorously, because the managerialist mindset comes with heightened expectations for individual agency, and an amalgamation between ideals of rational management and individual human rights (Bromley & Meyer, 2017; also see Hwang & Suárez, 2019). Increased reliance on practices for rational management and heightened expectations of individual rights and capacities are “twin cultural pillars” (Hwang & Suárez, 2019, p. 940).

Based on this theorization, we would expect that organizations that use more managerial practices also engage more strongly in advocacy, and hence also more strongly encourage their constituencies to engage in various forms of political activism. They would do so as vehicles in which individuals band together to pursue shared interests to assert them in a contested political arena. In fact, empirical studies that have examined the relationship between nonprofit managerialism, marketization, or being business-like, on the one hand, and nonprofit advocacy, on the contrary, have failed to find a negative relationship between these two sets of phenomena, and have often found a positive relationship (Hwang & Suárez, 2019; LeRoux & Goerdel, 2009; Lu, 2018; Suárez et al., 2018; Suykens et al., 2023). We therefore hypothesize:

**Hypothesis 2 (H2):** The use of managerial practices is positively related to the endorsement of public participation.

### Organizational Democracy and the Endorsement of Public Participation

Finally, regarding practices of organizational democracy in nonprofit organizations and the endorsement of public participation by these organizations, a positive relationship is to be expected. If decision-makers in an organization believe in the value of democracy at the organizational level—despite all the risks and cultural difficulties that entails—we can logically expect them to also value democracy at the societal level (Frega, 2020; Fung, 2003).

A qualitative study by Dodge and Ospina (2016) sheds light on the mechanisms underlying the positive link between organizational democracy and endorsement of public participation. They study two cases of community organizing nonprofits that engage their members in organizational decision-making, and in doing so also act as schools of democracy at the societal level. Organizations achieve this by framing their members as active citizens, focusing on accountability to members, giving them a voice, and fostering inclusion and diversity while building unity among their members. Quantitative evidence for a positive association between organizational democracy and public participation is provided by Fernandez et al. (2022), who—working with the concept of community and individual engagement—find that practices of organizational democracy correlate with promoting awareness of community-related issues and participation in community-related activities beyond the organization. Eikenberry (2022, p. 114) in her analysis of giving circles also provides quantitative evidence that in these nonprofit organizations, participatory practices at the organizational level go hand in hand with encouragement of civic and political engagement beyond the organization. We therefore hypothesize:

**Hypothesis 3 (H3):** The use of practices of organizational democracy is positively related to the endorsement of public participation.

**Figure 1.**
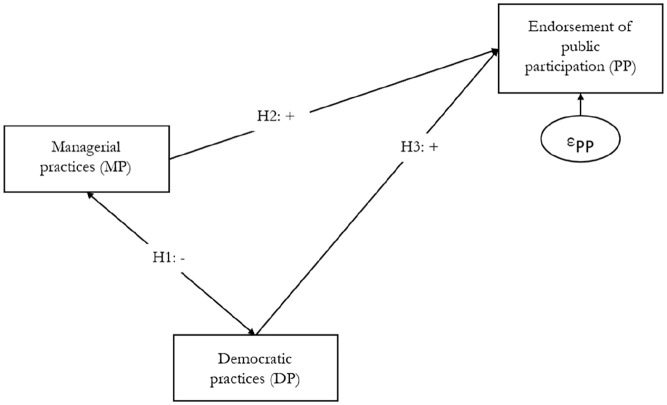
Research Model.

Figure 1 summarizes these hypotheses.

## Research Context and Methods^1^

### Research Context

We use survey data collected by the Civic Life of Cities Lab in an international collaboration exploring how nonprofit organizations in and around cities worldwide contribute to society. We focus on nonprofits in the metropolitan region of Vienna, Austria, which comprises some 2.6 million people living in three federal states (Vienna, Lower Austria, and Burgenland) and 211 municipalities.^2^

The nonprofit sector in that region is a sizable one: In 2017, when we drew the sample for our survey, Vienna had approximately 22,000 associations, 282 nonprofit corporations, 29 nonprofit co-operatives, and 121 nonprofit foundations, according to the relevant registers of administrative data (*Vereinsregister*, *Firmenbuch*, *Stiftungs- und Fondsregister*). In other words, there was one nonprofit organization for every 116 inhabitants.

Civil society in Vienna is characterized by a heavy reliance on membership fees, substantial government funding, nonprofit service providers and advocacy organizations, the important role of volunteers in all nonprofit fields, and the promotion of integration and peaceful coexistence of different ethnic and religious groups (Maier et al., 2022).

Nonprofit organizations in the region enjoy a high degree of civil liberties, as Austria—while not exempt from the global decline of democracy—is still one of the world’s most intact liberal democracies (Civicus, 2024). Although Austrian civil society has a troubled history, with uncivil elements supporting authoritarian forms of government in the past (Maier et al., 2022), since the end of World War II the overwhelming majority of nonprofit organizations have supported the democratic political system. While there are many conservative nonprofits, the extreme right is hardly present in the sector. This was evident, for example, in the presidential election the year after the refugee crisis in 2015, which came down to a runoff between a far-right candidate with illiberal ambitions, and a liberal-democratic opponent. This election was won by a razor-thin margin in favor of the liberal-democratic candidate, not least thanks to the broad mobilization efforts of nonprofit organizations of various stripes (Friedrich, 2016).

### Data Collection

The data used to test our hypotheses were collected through an online survey of nonprofit organizations in the Vienna Metropolitan Area from November 2019 to December 2020. A total of 593 organizations completed the survey, resulting in a response rate of 53% of the 1,117 organizations initially sampled (Table 1). For the model presented in this article, we were able to use 399 cases that answered all the questions used in the model. No missing values were imputed.

**Table 1.** Sample Descriptives, n = 399 (I).

ICNPO Categories	Frequency	Valid %
1100 Culture and Arts	49	12%
1200 Sports	60	15%
1300 Other Recreational and Social Clubs	26	6%
2000 Education and Research	48	12%
3000 Health	24	6%
4000 Social Services	62	15%
5000 Environment/Animal Protection	14	3%
6000 Development and Housing	35	9%
7000 Law, Advocacy and Politics	21	5%
9000 International	14	3%
10 000 Religion	9	2%
11 000 Business and Professional Associations, Unions	37	9%
12 000 Other, not elsewhere classified	0	0%
Total	399	100

The survey serves various research projects and, thus, includes a wide range of questions (see full questionnaire in the appendix). Survey items (questionnaire p. 7) allow us to identify organizations’ endorsement of public participation as well as their degree of organizational democracy (p. 3) and use of managerial practices (p. 13f). Furthermore, information on organizational characteristics such as membership figures, budget size, and age of the organization was collected.

Organizations fulfilling the nonprofit definition of Salamon and Sokolowski (2016) were eligible for participation, that is, self-governed private organizations with a limited profit-distribution requirement and non-compulsory participation. Pure grant-making foundations were not included. The sampling was carried out in two steps: (a) a random, representative sample from all nonprofit organizations in the region and (b) a random sample of large nonprofit organizations (annual budget of € 25,000 or more) in the region. In all, 358 organizations answering the survey belong to the representative sample and 235 to the sample of large organizations. Large organizations were oversampled because the majority of nonprofits in the region do not use many managerial practices and are very small (with only 36.1% having an annual budget of at least 25,000 EUR). To study the implications of managerial practices, sufficient variance in the data regarding the use of these practices had to be ensured (De Leeuw et al., 2012).

The survey was addressed to leaders of the organizations, such as CEOs or executive directors. Some of the organizations were led by an egalitarian team of board members. In these cases, it was left up to them which board member would answer the survey. The majority of respondents completed the survey online, and about a fifth of respondents requested to fill it in with the help of a researcher via telephone, video call, or in person. Respondents could choose to fill in the survey in either German or English.

To evaluate whether the final sample reflects the total population, we compared the distribution of fields of activity (NPOs were manually assigned to one field of activity as defined in the International Classification of Nonprofit Organizations, ICNPO, Salamon & Anheier, 1996) in the sample with the total population of organizations (Litofcenko et al., 2020). The Pearson chi-square test revealed that the structure of the responses does not deviate significantly from the structure of the population at the 95% significance threshold.

The NPOs in the sample of valid cases included in the model presented here vary in size, field of activity, age, and location. These organizations come primarily from four major fields: sports (15%), social services (15%), culture and arts (12%), and education and research (12%). Table 1 and Table 2 further describe these organizations according to their field of activity (ICNPO), size, and age. In all, 83% of the organizations were membership organizations, and 68% of the organizations involved volunteers.

**Table 2.** Sample Descriptives, n = 399 (II).

Variables	Frequency	Mean	Median	S.D.	Min	Max
Size (annual budget, in €)	399	7,442,330	120,000	46,477,440	0	800,000,000
Age (in years)	399	37.4	29.0	26.8	2.0	137.0

### Measures

To operationalize managerial practices, like previous quantitative studies using this concept (Brandtner, 2021; Suykens et al., 2023), we built on conceptualizations of managerialism (Maier & Meyer, 2011) and organizational rationalization (Hwang & Powell, 2009), and used the same or similar indicators: designated positions (paid or volunteer) for management and/or fundraising, working with consultants for management and/or fundraising, training of paid or volunteer staff in management and/or fundraising, having a mission statement, written budget plan, written strategic plan, and financial audit by an external professional auditor (questionnaire p. 13–15). These were scaled as dichotomous variables, and the mean over all items was calculated. Thus, the resulting managerial practices index ranges from 0 to 1.

Practices of organizational democracy were operationalized based on the following six items, also scaled as dichotomous variables (c.f., Maier & Meyer, 2011; Weber & Unterrainer, 2012; questionnaire p. 3): Whether people from the target group can participate in meetings of the board or other committees, have access to minutes from meetings, have the possibility to become a member to gain rights of co-determination; whether members or volunteers routinely initiate new projects, select executive staff, or are involved in communication to the general public. Again, the resulting index ranges from 0 to 1.

To capture the degree to which the organization endorses public participation, we combined the following eight dichotomous items (questionnaire p. 7): Whether the organization has encouraged employees, members, volunteers, or the target group during the last 3 years to vote in elections, to run for public offices, to attend public meetings (e.g., townhall meetings), to contact politicians, to participate in a rally, to organize a rally, to sign a petition, or to boycott specific brands or products. The resulting composite index ranges from 0 to 1. We had originally intended to discriminate between traditional and non-traditional forms of public participation. However, factor analyses and inter-item-correlations revealed that this distinction could not be upheld based on statistical properties (Allen & Yen, 2001). Rather, only one latent construct of public participation emerged from the data, in which five items originally intended to be included were not further used because they did not measure what they were intended to measure.

As all indicators used are based on factual data, that is, “data that is in principle verifiable from other sources” (Podsakoff & Organ, 1986, p. 532), we model our constructs with sum indices. With factual data, unlike subjective assessments, neither measurement errors nor common method bias pose an issue for the analysis (Podsakoff & Organ, 1986). Table 3 gives an overview on the descriptives of the constructs.

**Table 3.** Descriptives of Constructs (Valid Cases as Included in the Model, n=399).

	Mean	Correlation		
Constructs		Man.	Org.dem.	Pub. part.
Managerial practices (ω_3_=0.791, AVE=0.584)^a^	0.56	1		
Management/fundraising positions	0.52			
Consultants management/fundraising	0.40			
Trainings in management/fundraising	0.41			
Mission statement	0.62			
Budget plan	0.74			
Strategic plan	0.64			
External financial audit	0.57			
Organizational democracy (ω_3_=0.739, AVE=0.452)^a^	0.40	-0.16	1	
Participation option: access minutes	0.27			
Participation option: attend meetings	0.42			
Participation option: become a member	0.55			
Members/volunteers initiate new projects	0.47			
Members/volunteers select new executive staff	0.33			
Members/volunteers involved in public communication	0.37			
Endorsement of public participation (ω_3_=0.839, AVE=0.622)^a^	0.21	0.15	0.20	1
Endorse voting in elections	0.31			
Endorse running for public office	0.12			
Endorse attending public meetings	0.19			
Endorse contacting politicians	0.38			
Endorse participating in a rally	0.22			
Endorse organizing a rally	0.10			
Endorse signing petitions	0.31			
Endorse boycotting brands/products	0.09			

a As items are categorical, the hierarchical omega, ω_3_, is used to assess the convergent validity of the main constructs (Peters, 2014). All three constructs have ω_3_ above .7, thus reliability is satisfactory. To assess discriminant validity, we used the Fornell-Larcker criterion, which compares the average variance extracted (AVE) with the squared correlation of constructs (Fornell & Larcker, 1981). AVE should be greater than .5 and its square root should be greater than the correlations between the constructs. Managerial practices and endorsement of public participation pass the Fornell-Larcker criterion, organizational democracy is just below the threshold of .5. Despite this limitation, the discriminant validity of the constructs is supported, as the highest squared correlation between all pairs of constructs is less than the AVE of each construct in the respective pair.

### Methods of Analysis

To test our hypotheses, we implemented a path model with maximum likelihood estimation, using the open-source R-package *lavaan*. We controlled for the field of activity that organizations are primarily involved in and that is likely to affect the endorsement of public participation. Organizations in expressive fields of activity can be expected to more intensely endorse public participation (Frumkin, 2002; Jo, 2020; Putnam, 2007; Skocpol, 2003). Specifically, we categorized organizations belonging to ICNPO categories 5000 (environment and animal protection) and 7000 (law, advocacy, and politics) as those in expressive fields of activity.

## Findings

**Figure 2.**
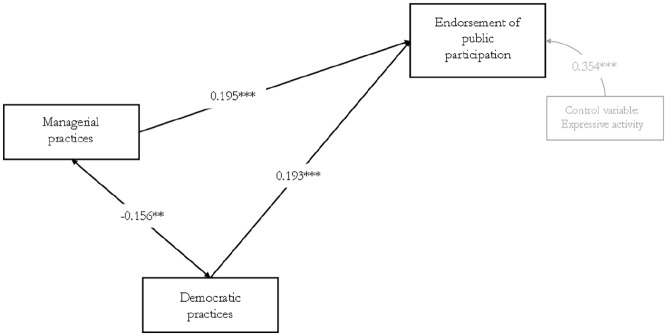
Standardized Parameter Estimates. *Note.* p-value (χ^2^): 0.0261; SRMR: 0.030; CFI: 0.992; TLI: 0.976. **p<.05; ***p<.01.

The results of the model (Figure 2) indicate a significant, albeit small, negative association (β=-0.156, *p*<.05) between nonprofits’ use of managerial practices and practices of organizational democracy. Thus, we find support for our first hypothesis. Nonprofits that use more managerial practices also endorse significantly more forms of public participation, although the effect is also small (β=0.195, *p*<.01). This supports our second hypothesis. Furthermore, in support of our third hypothesis, we find that nonprofits that use more organizational democracy practices endorse significantly more forms of public participation, but again the effect is small (β=0.193, *p*<.01). Finally, as expected, we find that organizations primarily engaged in expressive fields of activity endorse more forms of public participation, indicating a medium effect size (β=0.354, *p*<.01), compared with organizations active in other fields.

We performed three different kinds of robustness tests. First, we modeled endogenous constructs as latent factors and estimated a structural equation model (SEM), analogous to the path model. The measurement model indicates suitable reliability for all constructs, based on the Fornell-Larcker-criterion (Fornell & Larcker, 1981) and the average variance extracted (AVE). As an estimation method for the SEM, we employ the diagonally weighted least squares, which is recommended when many indicators are categorical (Hoyle & Panter, 1995, p. 169). The global fit indices show a moderate fit for the structural model (comparative fit index: 0.945, root mean square error of approximation: 0.054, standardized root mean square residual: 0.106; c.f. Hu & Bentler, 1999; Marsh et al., 2005). All effect sizes shift from small to medium in the SEM, and the relationships between the constructs are almost identical to parameter estimates of the path model. This supports the robustness of our more conservative path model.

As a second robustness test, we re-ran the path model using data from the representative sample only, i.e., a sample consisting mostly of small organizations. The results were similar to the full sample, but the negative correlation between managerial and democratic practices was smaller and no longer significant. This suggests a loss of significance not only due to the reduction in sample size, but also due to the importance of size as a control variable.

Hence, as a third robustness check, we again used the full sample and extended the path model to control for the effects of organizational size (by including the natural logarithm of the yearly budget), organizational age (in years), different sources of funding (membership fees, earned income, government funding), and cooperation relationships (with public organizations, other nonprofit organizations, or businesses) on managerial and democratic practices. Doing so, we found some significant effects: Organizational size has a positive effect on the degree of managerial practices used, and a negative effect on the degree of democratic practices used. By controlling for size, the negative relationship between managerial and democratic practices becomes insignificant. Organizational age is significantly positively related to the use of democratic practices. Cooperating with public organizations and receiving government funding is significantly positively related to the use of managerial practices. Since the aim of this article is not to comprehensively explain levels of managerial or democratic practices in organizations but to explain how managerial practices and different ways of serving as a school of democracy relate to each other, we shall delve no deeper into issues concerning these additional controls.

## Discussion and Conclusion

In this study, we have used path modeling based on organizational-level survey data to illuminate the relationship between nonprofit organizations’ managerial and democratic practices, and their endorsement of public participation. With this analysis, we make both an empirical and a theoretical research contribution.

First, we contribute empirical evidence to the scholarly debate about wider societal implications of nonprofit organizations becoming more business-like. Our findings support the argument from critical management studies about a positive relationship between organizational democracy and organizations promoting civic engagement in the public sphere (Eikenberry, 2022; King & Griffin, 2019). Moreover, our findings reinforce concerns that, as nonprofit organizations become more managerialized, they tend to become less democratic internally, as many critical scholars have warned. As other critical arguments—that managerialism may lead to mission drift or commercialization—have yet to be confirmed by quantitative studies, and quantitative evidence to the contrary has been substantial (e.g., Hersberger-Langloh et al., 2021; Shirinashihama, 2019; Suykens et al., 2023), it is paramount to emphasize that while warnings about other risks of managerialism may have been exaggerated, the warning about threats to organizational democracy has withstood our quantitative empirical scrutiny. This weakening of organizational democracy in nonprofits is likely to diminish the transmission of civic values and skills that neo-Tocquevillians (such as Dodge & Ospina, 2016; Van Ingen & Van der Meer, 2016) have pointed out. This may contribute to the erosion of democracy at the state level.

Second, by distinguishing between different ways in which nonprofits can serve as schools of democracy, we have arrived at more nuanced empirical insights that allow us to flesh out theoretical explanations of the causal mechanisms at work. We have distinguished between an internal and an external way in which nonprofits can serve as a school of democracy. The internal way involves nonprofits themselves adopting democratic structures, engaging beneficiaries, volunteers, or members in important organizational decisions, and providing them with in-depth insights into organizational decision-making processes. Conversely, the external way involves encouraging individuals to engage in political activities beyond the organization, such as voting, pursuing political office, signing petitions, or participating in protests. We find that as nonprofits become more managerialized, they tend to increase their endorsement of political participation but decrease their internal organizational democracy. In other words, they walk the walk of democracy less and talk the talk of democracy more.

The seemingly contradictory finding makes sense if we assume, in line with institutional theory and especially world polity theory (Bromley & Meyer, 2017; J. W. Meyer & Jepperson, 2000), that the managerialization of nonprofit organizations is part of the spread of world culture, with its twin pillars of rationalization and empowered actorhood (Hwang & Suárez, 2019). The drive for rationalization is based on the assumption that social activities can and should be managed through the application of science-like principles. The drive for empowered actorhood is based on the assumption that individuals, organizations, and states have many rights and capacities as actors. This entails an endorsement of democracy. However, there is an internal contradiction in the ideological edifice of world culture at large, and hence also in the institutional logic of managerialism (Lerch et al., 2022). The contradiction is that while the ideal of democracy is held in high esteem, it is based on a notion of individual agency. The presumably universal rationalist technologies promoted by world polity, such as the managerial practices examined in this study, provide little guidance on how to move from the views of individual human actors advocating for their interests, and even the legitimate interests of worthy others (Hwang & Suárez, 2019), to binding decisions within a democratic collective. In particular, they fail when the problem is not how to aggregate individual preferences into a collective decision by means of a mathematical algorithm (which may be as simple as a majority vote or as complex as proportional ranked choice voting), or how to find scientific truths and empirical facts, or how to allocate goods using market prices, but how to reconcile divergent views on issues where empirical certainty is not possible—or at least not at a reasonable cost—and where different value judgments collide.

How to become a united collective actor while valuing the differences among individual actors is, however, a key issue in organizational democracy, as Dodge and Ospina (2016) have shown. It is also crucial in democracy at the state level, as testified in mottos such as the United State’s *e pluribus unum* and the European Union’s *in varietate concordia*. Organizations that try to conform to cultural expectations about rational management find little guidance on how these expectations could be reconciled with empowering diverse groups in organizational decision-making (Mirabella et al., 2022). Knowledge about how to organize in managerial ways or how to organize in democratic ways has therefore coagulated into distinctive institutional logics (Knutsen, 2012). There is not much intuitive, commonsensical, readymade knowledge about how organizations can use managerial practices and be internally democratic at the same time (Baines et al., 2011; Hvenmark, 2013; Kreutzer & Jager, 2011). Our robustness checks involving control variables such as age and size of organization suggest that the larger an organization, the more difficult this problem becomes. This could be due to the fact that the more people are involved in organizational decision-making, the more differences need to be accommodated, and the more difficult it becomes to meet dominant cultural expectations of efficiency and speed.

Altogether, we thus find evidence for mechanisms of logic of appropriateness (March & Olsen, 2011) being at work here: Typically, highly managerialized nonprofit organizations just do what is widely deemed appropriate for organizations like them, namely, endorse democracy at the state level, and abstain from democratic experiments internally. The consideration of control variables in our empirical analysis shows that older organizations tend to be more democratic, regardless of their size and degree of managerialization. They may be able to draw on an older logic of appropriateness, as they carry the imprinting effect from their founding era when associations based on broad membership and methods of representative democracy were the norm in the Vienna metropolitan region (Maier et al., 2022). For them, it may be less of an experiment to combine democratic with managerial practices.

Consequently, the incompatibility between managerial practices and the practices of organizational democracy seems to be more a problem of limited imagination and lack of templates than one of actual technical incompatibility. This is testified by the exceptional cases in our data set, where when we look at organizations of any age and with any degree of managerial practices, we can find single cases of apparently thriving organizations that combine managerial practices with a high degree of organizational democracy.

From this, we conclude a main desideratum for further research on nonprofit organizations and management: There is need for more research on how democratic forms of organizing can be implemented in ways that satisfy key stakeholders while still allowing for the use of managerial practices that—as recent studies have shown (e.g., Hersberger-Langloh et al., 2021; Shirinashihama, 2019; Suykens et al., 2023)—fulfill not just legitimating but also legitimate purposes. With this recommendation, we are in line with the recent call “for organization” and for “putting management in its place” instead of being totally “against management,” as formulated by Parker (2023). Research should especially focus on the challenges and needs of larger nonprofit organizations, and of younger organizations that cannot simply refer back to historical patterns.

Our study has some limitations that must be noted and that also points to the need for further research. First, due to the nature of the survey data, we are restricted to cross-sectional data. Future research to test our hypotheses with longitudinal data would be valuable. Second, the survey data collected for this study is based on organizations as units of analysis. On the one hand, this provides a novel angle for the analysis of the schools of democracy or spillover effect, as most previous quantitative studies on this effect have used individual-level survey data (Timming & Summers, 2020; Van Ingen & Van der Meer, 2016; Weber et al., 2020). On the contrary, this means that we cannot draw definitive conclusions about the eventual effects of nonprofits’ endorsement of public participation. Specifically, we have no data on whether the individuals who were encouraged to engage in public participation indeed followed up with action. There has been some research suggesting that this link exists (LeRoux et al., 2023), but stronger evidence would be desirable. Combining organizational-level and individual-level data would allow for linking organizational behavior, individual perceptions, and public participation behavior in society. Finally, we use data from one region within a single country and hence cannot clearly take the influence of national political context into account. We studied nonprofit organizations in an old nonprofit sector with many organizations having a legacy of membership orientation and civic associational practices. The influence of isomorphic pressures toward managerialization has been relatively weak in this context, compared to Anglo-Saxon countries or very young nonprofit sectors in authoritarian contexts (e.g., the case of Shenzhen described by Long & Luo, 2022). Austria is also a country where democracy at the state level, while not entirely unscathed, is still highly intact, and one can confidently assume that the overwhelming majority of organizations that participated in our survey support the democratic political system, even if they represent a diverse range of political opinions. Research in more fragile democracies may come to different results or may have to use different interpretations of what endorsement of public participation means in those contexts. Further research to investigate the interplay between managerialism and nonprofit organizations’ role as schools of democracy (or schools of citizenship in an authoritarian context) in different political contexts would therefore be instructive.

Finally, we want to point out the practical implications of our findings. These concern nonprofit practitioners, but also scholars, educators, and trainers who teach nonprofit management and organizing. For nonprofit practitioners, we want to emphasize the need to value the role of nonprofit organizations as schools of democracy and to contribute to this role not only by endorsing democracy as a political system but also by practicing it internally. Democracy does not have to be antithetical to managerial virtues such as efficiency and speed, and sometimes slowing down and prioritizing resilience over efficiency might in any case be the better choice. Nonprofits need to be creative in finding ways to integrate organizational democracy with the demands of management, whether by rediscovering and rejuvenating traditions or by trying new approaches. For educators, we would argue that given the global crisis of democracy, we do not have time to wait another decade or two for a larger field of research to develop that will hopefully provide evidence of best practices in democratic organizing that meet the highest standards of scholarship. To put it bluntly, research on democratic organizing today is at a stage of development that research on leadership and management control was at a 100 years ago. We need to do our best at teaching democratic organizing now, based on the knowledge that is available now, while at the same time advancing research. This will be a key to overcoming the false but reality-defining dichotomy in people’s minds that management tools and organizational democracy are technically incompatible.

## Supplemental Material

sj-pdf-1-nvs-10.1177_08997640241278634 – Supplemental material for Talking the Talk, or Walking the Walk? How Managerial Practices Relate to Nonprofit Organizations’ Role as Schools of Democracy Supplemental material, sj-pdf-1-nvs-10.1177_08997640241278634 for Talking the Talk, or Walking the Walk? How Managerial Practices Relate to Nonprofit Organizations’ Role as Schools of Democracy by Lisa Hohensinn, Julia Litofcenko, Florentine Maier and Leila Cornips in Nonprofit and Voluntary Sector Quarterly

## Notes

1. We thank Berta Terzieva for allowing us to build on her descriptions of the research context and methods of data collection.

2. The delineation of the Vienna metropolitan region was based on the concept of Stadtregionen (Wonka & Laburda, 2010). Population data retrieved from the Stadtregionen Project Website: https://www.stadtregionen.at/wien.
