# Supplementary material for: Talking the Talk, or Walking the Walk? How Managerial Practices Relate to Nonprofit Organizations’ Role as Schools of Democracy
Source: Nonprofit Volunt Sect Q. 2024 Sep 20;54(4):749–72. doi: 10.1177/08997640241278634 (PMC12255473; doi:10.1177/08997640241278634)
Supplement: sj-pdf-1-nvs-10.1177_08997640241278634 – Supplemental material for Talking the Talk, or Walking the Walk? How Managerial Practices Relate to Nonprofit Organizations’ Role as Schools of Democracy [file sj-pdf-1-nvs-10.1177_08997640241278634.pdf]

English

Einleitung

Thank you for participating in the survey on "civil society in metropolitan regions"!

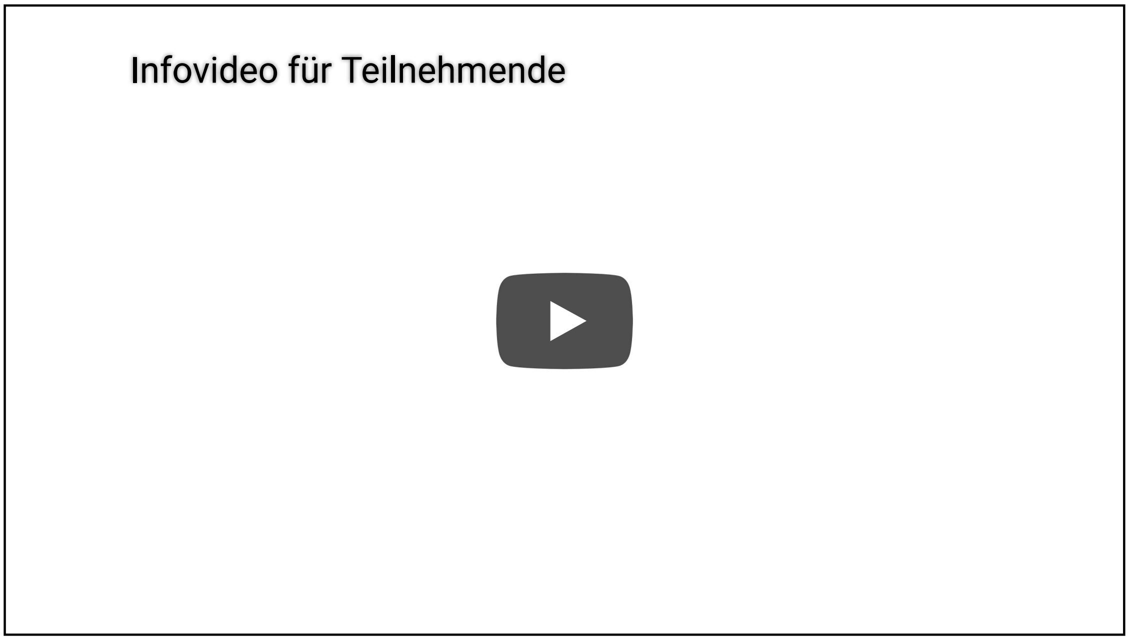

We protect your data.

We do not collect any personal data. We only ask questions about your personal experiences in an anonymous form. For statistical purposes, we record how long it takes to complete the various components of the survey. We only analyze the data in aggregate form, so that others will not be able to recognize you or your organization. We safeguard your rights guaranteed by the European General Data Protection Regulation and the EU Charter of Fundamental Rights. You can always stop the survey and ask us to delete your data.

We will be happy to send you an individualized report especially for your organization when the survey has been completed. If you would like this report, please tell us at the end of the survey.

Please do not hesitate to contact us if you have any questions or suggestions:
By telephone: 01/31336/5896
By mail: florentine.maier@wu.ac.at

Thank you very much for your cooperation!
PD Dr. Florentine Maier and Prof. Dr. Michael Meyer on behalf of the research team

Do you agree to participate in the survey?

- ○ I agree

Basisinformationen

What is the name of your organisation? (Please provide short name.)

[ ]

Die Ziele Ihrer Organisation

How do you typically refer to the target group(s) that your organization (${q://QID22/ChoiceTextEntryValue}) serves, engages with, or represents (e.g., clients, members, customers, patients, residents,...)?

[ ]

Please help us ask you appropriate questions by telling us:
Who is part of your target group (${q://QID286/ChoiceTextEntryValue})?

- ☐ People (e.g., individuals or groups of persons)
- ☐ Organizations
- ☐ Others (e.g., nature, animals in need)

Please imagine yourself in the position of a typical person from your target group.
How often does this person typically have personal contact with volunteers or paid staff from your organisation?

daily	weekly	monthly	yearly	irregularly	never
○	○	○	○	○	○

How well do the people from your target group and the volunteers/paid staff from your organisation **know each other**?

	practically everyone	more than half of them	about half of them	less than half of them	practically nobody
They greet each other on the street.	○	○	○	○	○
The recognize each other by name.	○	○	○	○	○
They spend time together outside of the organization's setting or activities.	○	○	○	○	○
They give each other advice about topics unrelated to the organization's activities.	○	○	○	○	○
They participate in each others' life events (e.g., attend birthdays, weddings).	○	○	○	○	○
	practically everyone	more than half of them	about half of them	less than half of them	practically nobody

What opportunities do people from your target group (${q://QID286/ChoiceTextEntryValue}) have to participate in the organization's decisions?

(Please select all that apply.)

- ☐ None
- ☐ Formally join the organisation (e.g., become a member) to have more participation rights
- ☐ Participate in public meetings of committees or board
- ☐ Access meeting minutes
- ☐ Visit us in person and give feedback
- ☐ Comment on documents and reports
- ☐ ________ Other opportunities:

Please think of 10 typical people from your target group (${q://QID286/ChoiceTextEntryValue}).

What is the age of the people in your target group?

	0	1	2	3	4	5	6	7	8	9	10	
30 years or younger												
31-64 years old												
65 years old or older												
don't know												

What is the first language of the people in your target group?

(First language = the language people learned first in their life)

	0	1	2	3	4	5	6	7	8	9	10	
German												
other first language(s) than German												
don't know												

What is the gender of the people in your target group?

	0	1	2	3	4	5	6	7	8	9	10	
female												
male												
don't know												

Please use this field if you would like to tell us more about the gender distribution of these people:

Where is your target group (${q://QID286/ChoiceTextEntryValue}) mostly located?

- In a certain district in Vienna, or in a certain municipality in Lower Austria:
- All over Vienna, or all over Lower Austria
- All over Austria
- All over Europe

○ Outside Europe

○ Other: ______

How have the following things changed in your organization over the past three years?

	decreased	stayed the same	increased	no answer possible
Demand for activities of the organization	○	○	○	○
In-person interactions with people from our target group	○	○	○	○
Virtual interactions with people from our target group	○	○	○	○

How important are the following activities to the mission of your organization?

	critical to mission	supports mission/ desired side effect	unimportant
Providing high-quality programs/services.	○	○	○
Trying out new approaches to programs/services.	○	○	○
Empowering people to stand up for their interests.	○	○	○
Speaking at public workshops, conferences, fora, etc. in the interest of our target group.	○	○	○
Providing programs/services at a subsidized prize.	○	○	○
Providing programs/services that have not been provided before for our target group.	○	○	○
Developing quality standards in our domain.	○	○	○

	critical to mission	supports mission/ desired side effect	unimportant
Meeting an unmet need for a particular program/service.	○	○	○
Providing cost-efficient programs/services.	○	○	○
Building trust between people.	○	○	○
Providing programs/services free of charge to all.	○	○	○
Providing a place for people to feel a sense of belonging.	○	○	○
Public education campaigns to raise awareness.	○	○	○
Issuing public statements and reports in the interest of our target group.	○	○	○

critical to mission supports mission/ desired side effect unimportant

	critical to mission	supports mission/ desired side effect	unimportant
Promoting regular interactions between people.	○	○	○
Providing a vehicle for private donors to express their values by supporting us.	○	○	○
Being a public voice for a particular group or topic.	○	○	○
Supporting special interests that receive little support from government or businesses.	○	○	○
Promoting civic education.	○	○	○

critical to mission supports mission/ desired side effect unimportant

In the past three years, has your organisation encouraged or discouraged staff, members, volunteers or your target group (${q://QID286/ChoiceTextEntryValue}) to do any of the following?

	encourage	discourage	weder zu- noch abgeraten
vote in elections	O	O	O
run for public office	O	O	O
start a new organization	O	O	O
volunteer for or join another organization	O	O	O
donate to or raise funds for another organization	O	O	O

	encourage	discourage	weder zu- noch abgeraten
organize a rally	O	O	O
participate in a rally	O	O	O
attend public meetings (e.g., town hall, city council meeting)	O	O	O
boycott particular brands or products	O	O	O
sign petition	O	O	O

	encourage	discourage	weder zu- noch abgeraten
contact politicians	O	O	O
discuss the organization’s cause with family or friends	O	O	O
write about the organization in social media (e.g., Facebook, Twitter)	O	O	O

Does your organization **host or sponsor any events** (e.g., charity events, festivals, conferences, public meetings, rallies, organization-sponsored volunteer work days)?

- O yes
- O no

Approximately how many events did your organization host or sponsor last year?

	Number of events
Recreational activities	0
Charity events or fundraisers	0
Festivals or celebrations	0
Conferences, lectures, panel discussions, seminars	0
Public meetings, hearings, petitions	0
Rallies, demonstrations, marches	0
Volunteer work days (e.g., cleaning days, visiting days)	0
Others: ______	0

Were the subjects of these events related to issue(s) facing...

(Check all that apply.)

- ☐ a particular Viennese district or municipality in Lower Austria
- ☐ all of Vienna, or all of Lower Austria
- ☐ all of Austria
- ☐ all of Europe
- ☐ the whole world
- ☐ ______ others:

Has your organisation been involved in **policy-making processes** over the last three years (e.g. giving feedback to legislative proposals, community participation, consultations)?

	routinely involved	occasionally involved	never involved
At the regional level (e.g., in a Viennese district, in a municipality in Lower Austria)	○	○	○

	routinely involved	occasionally involved	never involved
At the provincial level (e.g., city of Vienna, province of Lower Austria)	O	O	O
At the federal level in Austria	O	O	O
At the European level	O	O	O
At the global level	O	O	O
	routinely involved	occasionally involved	never involved

Ihr persönlicher Hintergrund und die Leitung der Organisation

Is your position in this organization paid, or are you a volunteer?

paid	partly paid, partly volunteer	volunteer
O	O	O

Do you work for the organization full-time?

Yes	No
O	O

Please tell us about your work experience outside the organisation (${q://QID22/ChoiceTextEntryValue}).
In which other jobs are you or have you been working?

Job title __________

Job title __________

Job title __________

Was/is this job in the **private sector** or in the **public sector** (government/state)?

	private	public (government/state)	don't know
» Job title	O	O	O
» Job title	O	O	O
» Job title	O	O	O

Was/is this job in a **profit** oriented organization or a **non-profit** oriented organization?

	for-profit	not-for-profit	don't know
» Job title	○	○	○
» Job title	○	○	○
» Job title	○	○	○

What formal education do you have?

(Please select all that apply)

- ☐ None
- ☐ Compulsory schooling completed
- ☐ High school degree
- ☐ Vocational training completed
- ☐ Academic degree(s) (e.g., bachelor, master, doctorate)

What kind of high school degree do you have?

AHS (high school for general education)	HTL (high school for technical professions)	HAK/HBLA (high school for commercial professions)	Other
○	○	○	○

What vocational training do you have?

____

What academic degree(s) do you have?

____

What is the term for the top leadership position in your organization (${q://QID22/ChoiceTextEntryValue})?

____

Has there been turnover in the top leadership position (${q://QID271/ChoiceTextEntryValue}) in the last three years?

	Change	No change	Can't answer (e.g., cannot recall, organization did not exist)
2018-2019	○	○	○

	Change	No change	Can't answer (e.g., cannot recall, organization did not exist)
2016-2017	○	○	○
2014-2015	○	○	○

Die Menschen in der Organisation

Does your organization have **members**?

○ [ ] **Yes.** The approximate **number** of our members is:

○ **No.** Membership does not play a role for our organization.

How many paid staff does your organization (${q://QID22/ChoiceTextEntryValue}) employ? (Please enter 0 if there are none.)

Full-time (ca 40 hours/week) [ ]

Part-time [ ]

How important is volunteer work (i.e., unpaid work by volunteers, board members or regular members) for your organization (${q://QID22/ChoiceTextEntryValue})?

very unimportant ○ ○ ○ ○ ○ very important

How many people volunteered in your organization (${q://QID22/ChoiceTextEntryValue}) last year?

(Type 0 if none.)

[0] People who engaged in one-off volunteering

[0] People who engaged in routine/regular volunteering

[0] Others: [ ]

Please think of **10 typical people** doing **volunteer or paid work** for your organization (${q://QID22/ChoiceTextEntryValue}).

What is the **age** of the people in that group?

(If fewer than 10 people do volunteer or paid work for your organization, please estimate the approximate

distribution.)

	0	1	2	3	4	5	6	7	8	9	10	
30 years or younger												☐
31-64 years old												☐
65 years old or older												☐
don't know												☐

What is the **first language** of these people?

(First language = the language that people learned first in life)

(If fewer than 10 people do volunteer or paid work for your organization, please estimate the approximate distribution.)

	0	1	2	3	4	5	6	7	8	9	10	
German												☐
other first language(s) than German												☐
don't know												☐

What is the **gender** of these people?

(If fewer than 10 people do volunteer or paid work for your organization, please estimate the approximate distribution.)

	0	1	2	3	4	5	6	7	8	9	10	
female												☐
male												☐
don't know												☐

Please use this field if you would like to tell us more about the gender distribution of these people:

☐

Have you or volunteers/staff members participated in training programs on any of the following topics as part of their work for the organization (${q://QID22/ChoiceTextEntryValue}) over the past three years?

(Please select all that apply.)

	You	volunteers/staff members
Topics specific the field of activity of your organization (${q://QID22/ChoiceTextEntryValue}) (Examples for topics specific to the field of activity: hospital - medical topics, kindertgarten - pedagogical topics)	☐	☐
Interpersonal skills	☐	☐
Advocacy	☐	☐
Information technology (e.g., social media, software, digital platforms, information security, data protection, data analytics)	☐	☐
Fundraising	☐	☐
Other management topics	☐	☐
Other: ____________	☐	☐
	You	volunteers/staff members

Over the past 3 years, has your organization had or changed **positions** responsible for any of the following tasks?

	already existed	created new	expanded existing	contracted
Tasks specific to the field of activity of your organization (${q://QID22/ChoiceTextEntryValue}) field of activity (Examples for tasks specific to the field of activity: hospital - medical tasks, kindertgarten - pedagogical tasks)	☐	☐	☐	☐
General leadership tasks	☐	☐	☐	☐
Advocacy	☐	☐	☐	☐
Information technology (e.g., social media, software, digital platforms, information security, data protection, data analytics)	☐	☐	☐	☐
Fundraising	☐	☐	☐	☐
Other managerial tasks	☐	☐	☐	☐

	already existed	created new	expanded existing	contracted
Other	☐	☐	☐	☐
	already existed	created new	expanded existing	contracted

Over the past 3 years, has your organization brought in consultants to advise your organization on any of the following topics?

	YES, we paid for them	YES, somebody else paid for them	YES, they worked for us for free
Topics specific to your organization's (${q://QID22/ChoiceTextEntryValue}) field of activity (Examples for topics specific to the field of activity: hospital - medical topics, kindertgarten - pedagogical topics)	☐	☐	☐
Interpersonal skills	☐	☐	☐
Advocacy	☐	☐	☐
Information technology (e.g., social media, software, digital platforms, information security, data protection, data analytics)	☐	☐	☐
Fundraising	☐	☐	☐
Other managerial topics	☐	☐	☐
Other:	☐	☐	☐
	YES, we paid for them	YES, somebody else paid for them	YES, they worked for us for free

Organisation und Management

Does your organization have any of the following?

	YES, for the entire organization	NO, not any more	No, we never have
Publicly available report about the organization's activities (e.g., annual report)	○	○	○
Exchange with external experts about current developments in our area of expertise	○	○	○

	YES, for the entire organization	NO, not any more	No, we never have
Ceremonies or celebrations<br>by members, volunteers and/or staff in the context of the organization	O	O	O
Mission statement	O	O	O

	YES, for the entire organization	NO, not any more	No, we never have
Evaluation of the organisation's activities with methods recognised in our area of expertise	O	O	O
Systematic collection of information about about the wishes of people from our target group<br>(e.g., survey, focus group)	O	O	O
Strategic plan<br>(=a document about what your organization wants to achieve, and how it will do so)	O	O	O
Written budget plan	O	O	O

	YES, for the entire organization	NO, not any more	No, we never have
Financial audit by external professional auditor	O	O	O
Conveying of founding history of the organization<br>to members, volunteers and/or staff	O	O	O
Group meals, chats, or social events<br>of members, volunteers and/or staff as part of their work for the organization	O	O	O

Are there any other procedures or tools that are essential for the success of your organization (${q://QID22/ChoiceTextEntryValue})?

O [ ] Yes (please specify)

○ No

We would like to know how your organization engages with technology. Which of the following do you have?

	YES, we have	NO, but we are currently considering or developing	NO, not anymore	NO, not an issue for us
Software for managing staff or productivity	○	○	○	○
Own social media presence (e.g. on Facebook, Twitter)	○	○	○	○
Own website	○	○	○	○
Formal policy on data privacy or data use	○	○	○	○
Own app	○	○	○	○
Own mailing list	○	○	○	○
Database of people from our target group or donors	○	○	○	○
Online service provision	○	○	○	○
Internal communication software (e.g., with a wiki, file sharing, whiteboard, meetings function)	○	○	○	○
	YES, we have	NO, but we are currently considering or developing	NO, not anymore	NO, not an issue for us

Who is routinely involved in the following tasks?

	Top leader	board	paid staff	volunteers	members	people from our target group	external consultants	others
Contributing content to website	☐	☐	☐	☐	☐	☐	☐	☐

	Top leader	board	paid staff	volunteers	members	people from our target group	external consultants	others
Developing the strategic plan	☐	☐	☐	☐	☐	☐	☐	☐
Develop ideas for new programs/services to be offered by the organization	☐	☐	☐	☐	☐	☐	☐	☐
Selecting people for leadership positions	☐	☐	☐	☐	☐	☐	☐	☐
Creating publicly available report about the organization's activities	☐	☐	☐	☐	☐	☐	☐	☐
Planning the budget	☐	☐	☐	☐	☐	☐	☐	☐
Developing the mission statement	☐	☐	☐	☐	☐	☐	☐	☐
Contributing content to social media	☐	☐	☐	☐	☐	☐	☐	☐
	Top leader	board	paid staff	volunteers	members	people from our target group	external consultants	others

Erfolgsmessung

Please briefly explain your organization's mission.

[ ]

From your perspective, what are the most important indicators that let you know whether your organization is being successful?

Indicator 1 [ ]

Indicator 2 [ ]

Indicator 3 [ ]

How successful would you rate your organization on the basis of these indicators?

	much worse	a bit worse	about the same	a bit better	much better
Compared to your organization three years ago	O	O	O	O	O
Compared to similar organizations	O	O	O	O	O

How does your organization monitor or evaluate whether it is successful?

- ☐ We do not monitor or evaluate that.
- ☐ Listening to the people involved.
- ☐ Participation in competitions
- ☐ External certifications, accreditations and/or seals of quality
- ☐ External ratings or rankings
- ☐ Written surveys of the target group about their satisfaction
- ☐ Input metrics (e.g., how many working hours or how much money went into a particular offer by the organization)
- ☐ Output metrics (e.g., how many people made use of a particular offer by the organization)
- ☐ Impact metrics (e.g., whether a particular offer by the organization had a positive effect on the users' behaviour)
- ☐ ________ Other:

Finanzielles

We would like to have a rough idea about the financial situation of your organization.

This data will help us understand the circumstances of your organization. We will treat your data confidentially. All analyses will be based on overall statistics, without naming your organisation.

Estimates are sufficient for the following questions. You don't have to get out your accounting documents for this!

What was your organization's **budget** last year?
A rough estimate is sufficient.

(In other words: How much money did your organisation have available or spend last year?)

[0] EUR

What proportion of your funding came from these sources?

	0%	100%	
	0	100	
Donations from private individuals			0
Donations from businesses			0
Earned income from selling services/products			0
Government			0
Membership fees			0
Foundations			0
Other (e.g., rent, sales of assets) [ ]			0

How much of the earned income from selling services/products came from what kind of activities?

	0%	100%	
	0	100	
Activities that **in themselves** contribute to our mission (e.g., selling products from a work integration social enterprise)			0
Activities that **only through the achieved revenue** contribute to our mission. (e.g., selling parking tickets for garage)			0
Other kinds of activities			

0% 100% 0

0 100

How much of the total money you had available last year came from the following regions?

	much	some	nothing
from a particular Viennese district, or from a particular municipality in Lower Austria	○	○	○
from the whole city of Vienna, or from the whole of Lower Austria	○	○	○
from the whole of Austria	○	○	○
from the whole of Europe	○	○	○
from outside Europe	○	○	○
	much	some	nothing

Is your organization using or considering any of the following for **funding**?

	YES, using	YES, considering	NO, not using or considering	Never heard of it
crowdfunding	○	○	○	○
social impact bonds	○	○	○	○
impact investments (= Somebody invests into your organization at market-rate returns)	○	○	○	○
earned income from selling services/products	○	○	○	○
program-related investments (= Somebody invests into your organization at below-market returns)	○	○	○	○
venture philanthropy (= Somebody invests into a project of your organization and also supports it by providing advice and contacts)	○	○	○	○

	YES, using	YES, considering	NO, not using or considering	Never heard of it
taking donations in cryptocurrencies	O	O	O	O
associated for-profit venture	O	O	O	O
	YES, using	YES, considering	NO, not using or considering	Never heard of it

Imagine you stopped receiving funding today.

How many months could your organization continue to work as before?

[ ]

Approximately what proportion of your annual budget is used for...

(drag the sliders to the approximate values; must sum to 100%)

	0	10	20	30	40	50	60	70	80	90	100	
Facilities or space (e.g., rent)												0
Compensation for employees (e.g., salaries)												0
All other expenses												0

Beziehungen zu anderen Organisationen

How would you describe your organization’s status?

(Check all that apply.)

- ☐ We are an entirely independent organization.
- ☐ We are the headquarters or umbrella organization of associated nonprofit organizations.
- ☐ We are the "daughter" of a larger nonprofit organization
- ☐ We are the nonprofit "daughter" of a for-profit business
- ☐ We are the nonprofit "daughter" of a governmental agency or public organization
- ☐ [ ] Other

Is your organisation a member of umbrella organisations, platforms, organisational networks, etc?

If so, of which?

Examples: umbrella organisations in your field of activity (e.g. Austrian Federal Sports Organisation, Lower Austrian Association of Parents' Associations), Interest Group of Non-Profit Organisations, NPO Institute, Association of Catholic Associations,...

- ○ Yes (please specify) [ ]
- ○ No

Does your organization collaborate with any of the following organizations for any of the following purposes?

	with for-profit businesses	with government agencies/public organizations	with other nonprofit organizations	with foundations
delivering services for our target group	☐	☐	☐	☐
advocacy	☐	☐	☐	☐
building community between people	☐	☐	☐	☐
building the capacities of our organization	☐	☐	☐	☐
commercial purposes	☐	☐	☐	☐
recruiting volunteers	☐	☐	☐	☐
organizing events	☐	☐	☐	☐
	with for-profit businesses	with government agencies/public organizations	with other nonprofit organizations	with foundations

Are there any organizations with whom your organizations has a particularly strong relationship?

- ○ Yes (which ones) [ ]
- ○ No

You have not reported any collaborative relationships of your organization. Are there any other ways in which you interact with other organizations?

- ○ No
- ○ [ ] Yes:

Are there other organisations that provide similar offers as your organisation for exactly the same target group?

- ☐ [ ] Private for-profit businesses (please specify)
- ☐ [ ] Government organizations/public agencies (please specify)
- ☐ [ ] Other nonprofits (please specify)
- ☐ [ ] Foundations (please specify)
- ☐ [ ] Others:

Schluss

Thank you!

Is there anything else you would like to tell us about this survey?

[ ]

Would you like to see your answers in comparison to the overall statistics?

In that case please give us an e-mail address.

We will send you an individualized report for your organization when all the data has been collected.

E-mail address: [ ]

May we contact you by telephone if we have any questions?
If you agree, please give us your telephone number:

Powered by Qualtrics
